# MOG-IgG-Associated Optic Neuritis, Encephalitis, and Myelitis: Lessons Learned From Neuromyelitis Optica Spectrum Disorder

**DOI:** 10.3389/fneur.2018.00217

**Published:** 2018-04-04

**Authors:** Giordani Rodrigues dos Passos, Luana Michelli Oliveira, Bruna Klein da Costa, Samira Luisa Apostolos-Pereira, Dagoberto Callegaro, Kazuo Fujihara, Douglas Kazutoshi Sato

**Affiliations:** ^1^School of Medicine, Brain Institute of Rio Grande do Sul (BraIns), Pontifical Catholic University of Rio Grande do Sul (PUCRS), Porto Alegre, Brazil; ^2^Department of Neurology, Hospital das Clínicas from the Faculty of Medicine, University of São Paulo (HC-FMUSP), São Paulo, Brazil; ^3^Department of Multiple Sclerosis Therapeutics, Multiple Sclerosis and Neuromyelitis Optica Center, Southern TOHOKU Research Institute for Neuroscience, Fukushima Medical University, Koriyama, Japan

**Keywords:** neuromyelitis optica spectrum disorder, optic neuritis, myelitis, encephalitis, myelin oligodendrocyte glycoprotein antibody

## Abstract

Antibodies against myelin oligodendrocyte glycoprotein (MOG-IgG) have been found in some cases diagnosed as seronegative neuromyelitis optica spectrum disorder (NMOSD). MOG-IgG allowed the identification of a subgroup with a clinical course distinct from that of NMOSD patients who are seropositive for aquaporin-4-IgG antibodies. MOG-IgG is associated with a wider clinical phenotype, not limited to NMOSD, with the majority of cases presenting with optic neuritis (ON), encephalitis with brain demyelinating lesions, and/or myelitis. Therefore, we propose the term MOG-IgG-associated Optic Neuritis, Encephalitis, and Myelitis (MONEM). Depending on the clinical characteristics, these patients may currently be diagnosed with NMOSD, acute disseminated encephalomyelitis, pediatric multiple sclerosis, transverse myelitis, or ON. With specific cell-based assays, MOG-IgG is emerging as a potential biomarker of inflammatory disorders of the central nervous system. We review the growing body of evidence on MONEM, focusing on its clinical aspects.

## Introduction

Antibodies against myelin oligodendrocyte glycoprotein (MOG-IgG) have been found in certain cases diagnosed as seronegative neuromyelitis optica spectrum disorder (NMOSD). NMOSD is an inflammatory condition of the central nervous system (CNS), mainly characterized by optic neuritis (ON) and transverse myelitis (TM) and encompassing the entity previously known as neuromyelitis optica (NMO) along with limited forms of the disease; these entities are now unified under the term NMOSD (1).

Neuromyelitis optica spectrum disorder is traditionally associated with attacks on the optic nerves and spinal cord, and the discovery of an NMO-specific antibody in 2004 (2) allowed these patients to be distinguished from those with multiple sclerosis (MS). One year later, aquaporin-4 was identified as its target antigen, present on the end-feet processes of astrocytes (3). Until 2015, positivity for aquaporin-4 immunoglobulin G (AQP4-IgG) suggested NMO, although some patients with the classic NMO phenotype remained seronegative for AQP4-IgG despite the use of increasingly sensitive serologic assays (4–6). Furthermore, several patients with limited forms of the disease, such as isolated ON or TM, or extra-optic-spinal involvement, such as area postrema and brain lesions, were AQP4-IgG positive, further challenging the traditional definition of NMO.

In this context, we and others have found the presence of autoantibodies against myelin oligodendrocyte glycoprotein (MOG-IgG) in some patients with clinical presentations suggestive of NMOSD (5, 7–9). However, MOG-IgG is associated with a wider clinical phenotype, not limited to NMOSD, and only a third of MOG-IgG-seropositive patients or fewer fulfill the current diagnostic criteria for NMOSD (10–13).

**Figure 1:**
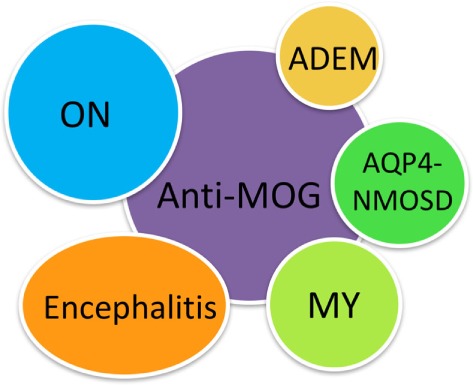
MOG-IgG-associated optic neuritis, encephalitis, and myelitis (MONEM). The clinical phenotypes associated with MOG-IgG are encompassed under the term MONEM. MONEM is not limited to aquaporin-4-IgG-negative neuromyelitis optica spectrum disorder (NMOSD).

The majority of MOG-IgG-seropositive cases have ON, encephalitis with brain demyelinating lesions, and/or myelitis; thus, we propose the new term MOG-IgG-associated ON, encephalitis, and myelitis (MONEM) to encompass this group of patients with CNS demyelinating syndromes associated with MOG-IgG (Figure 1). Depending on the clinical assessment, patients can currently be clinically diagnosed with NMOSD, acute demyelinating encephalomyelitis (ADEM), pediatric MS, or isolated myelitis or ON (14, 15).

Recently, multiple research groups have described isolated cortical and subcortical syndromes in MOG-IgG-seropositive patients, highlighting the importance of encephalitis in this context (16–20). Furthermore, MOG-IgG acute encephalitis is associated with pathological evidence of severe demyelination without the astrocyte loss usually recognized in AQP4-IgG-seropositive NMOSD cases (21). MOG-IgG-associated encephalomyelitis and isolated encephalitis represent important clinical syndromes associated with MOG-IgG and may be a bridging element in the emerging spectrum of MOG-IgG-associated syndrome. Currently, it remains unclear whether MONEM should indicate a different disease. We review the growing body of evidence on MOG-IgG in NMOSD and other CNS inflammatory diseases, focusing on its clinical aspects, and we report a clinical vignette of a MOG-IgG-positive patient fulfilling the NMOSD criteria to highlight some of the features and challenges of this condition.

## MOG-IgG: Pathophysiological Aspects

MOG, a glycoprotein of the immunoglobulin superfamily, is a component of the CNS myelin sheath, as are myelin basic protein (MBP) and proteolipid protein (22). The precise functions of MOG remain to be elucidated but likely include roles in the adhesion of myelin fibers, regulation of oligodendrocyte microtubule stability, and modulation of the interaction between myelin and the immune system by the complement pathway (23, 24).

Even though MOG is a minor component of the CNS myelin sheath, accounting for less than 0.5% of its composition, many of its epitopes have been demonstrated to be highly immunogenic, both in rodents and humans (25–27).

Immunization of rodents with MOG-derived antigens (28–30) and generation of rodents with transgenic MOG-specific T cell receptors (31) produce CNS lesions that resemble some of the features of MS and NMOSD. Notably, double-transgenic mice with both MOG-specific T cell receptors and an MOG-specific immunoglobulin heavy chain present more severe experimental autoimmune encephalitis than single transgenic mice, revealing that T cell/B cell cooperation is a key aspect in the pathogenesis of CNS autoimmunity (32, 33).

In humans, high-titer MOG-IgG in serum samples seems to efficiently activate the complement cascade *in vitro* (9). Moreover, purified IgG from MOG-IgG-seropositive patients, when incubated with oligodendrocytes *in vitro*, led to marked disorganization of the cytoskeleton, further suggesting functional pathogenicity (34).

Despite the fact that MONEM can overlap with the clinical presentation of AQP4-IgG-associated NMOSD, the mechanisms that underlie MOG-IgG-driven diseases are likely different. Indeed, several animal and human studies have revealed immunologic and pathological differences between AQP4-IgG NMOSD and MONEM, suggesting that these two groups are different nosological entities.

From a pathological standpoint, there are striking differences between MOG-IgG and AQP4-IgG groups. Whereas the pathological hallmark of AQP4-IgG NMOSD is astrocytic damage, with secondary oligodendrocyte loss and demyelination (35), no evidence of astrocytopathy has been reported in MOG-IgG cases. In support of that view, a case with an AQP4-seronegative MOG-IgG-seropositive NMO phenotype in which cerebrospinal fluid (CSF) examination showed elevated MBP, in the absence of detectable glial fibrillary acidic protein (GFAP), has been reported (36). This distinction has been further validated by a multicenter CSF study indicating elevated MBP in MOG-IgG patients without elevation of GFAP compared with AQP4-IgG-seropositive NMOSD (37). These findings suggest inflammation and myelin destruction without astrocyte injury, thus making the CSF profile of MONEM clearly different from that of AQP4-IgG-associated NMOSD. Therefore, we avoid the term “MOG-IgG-associated NMOSD,” as the current understanding of NMOSD (at least in those patients with AQP4-IgG) indicates an immune-mediated disorder targeting astrocytes, which contrasts with MONEM, a demyelinating disorder affecting oligodendrocytes (38). Of note, however, it is still unknown whether the same astrocytopathic pattern holds true for AQP4-IgG-seronegative NMOSD.

So far, the histopathology associated with MOG-IgG in humans is based on only a few reported cases: two patients with recurrent longitudinally extensive transverse myelitis (LETM) plus tumefactive brain lesions (16, 21), two patients with ON plus clinical or subclinical brain involvement (39), two patients with ADEM (40), and one patient whose initial diagnosis was clinically isolated syndrome suggestive of MS (hemiparesis, impaired coordination, and headache) (41). Of note, yet compatible with MONEM, these are not the most common presentations, and the overrepresentation of such more severe phenotypes, with brain involvement, can be explained by indication bias for brain biopsy. In all cases, lesions have been described as clearly demyelinating, with marked infiltration of macrophages (often containing myelin degradation products) and T cells, and relative preservation of axons and astrocytes (16, 21, 39–41). In most cases, B cell infiltration (16, 39–41) and IgG and complement deposition (21, 40, 41) have been reported as well. Interestingly, the histopathological features above are compatible with the so-called pattern II lesions of MS (42). Apart from the cases above, another one has been described with fulminant encephalomyelitis, but with detection of both MOG-IgG (early in the disease course) and AQP4-IgG (later on); in this case, the histopathological aspect of lesions represented an overlap of features compatible with pattern II MS and features usually seen in AQP4-IgG-seropositive NMOSD (43).

## MOG-IgG Assays

Recent advances in the laboratory techniques used to detect MOG-IgG have impacted both the current approach used to determine serological status and our interpretation of findings from earlier studies, which used less accurate detection techniques. It has been demonstrated that the biologically relevant MOG antibodies are those recognizing conformational MOG epitopes (44, 45). However, the early studies used western blotting, which detects unfolded, denatured MOG protein, or ELISA (for linear peptides), which did not distinguish specific antibodies against conformational MOG epitopes (46).

The development of cell-based assays (CBA) using transfected cells enabled the identification of clinically relevant MOG-IgG (7, 47). However, even among studies that used CBA, technical heterogeneity remains an issue and may explain some variable or conflicting results. Moreover, it has been suggested that variations in the epitope specificity of human MOG-IgG (as indicated by the ability of patient serum to also recognize rat or mouse MOG, leading to different immunohistochemical staining patterns) may be associated with different clinical presentations (10). This variation may indicate that MOG-IgG has different effects depending on the antibody characteristics (e.g., affinity, capacity to activate the complement system) and the downstream effects on oligodendrocytes after the binding of MOG-IgG to MOG on the surface of the myelin sheath.

## MOG-IgG in Clinically Suspected NMOSD and Related Disorders

**Figure 2:**
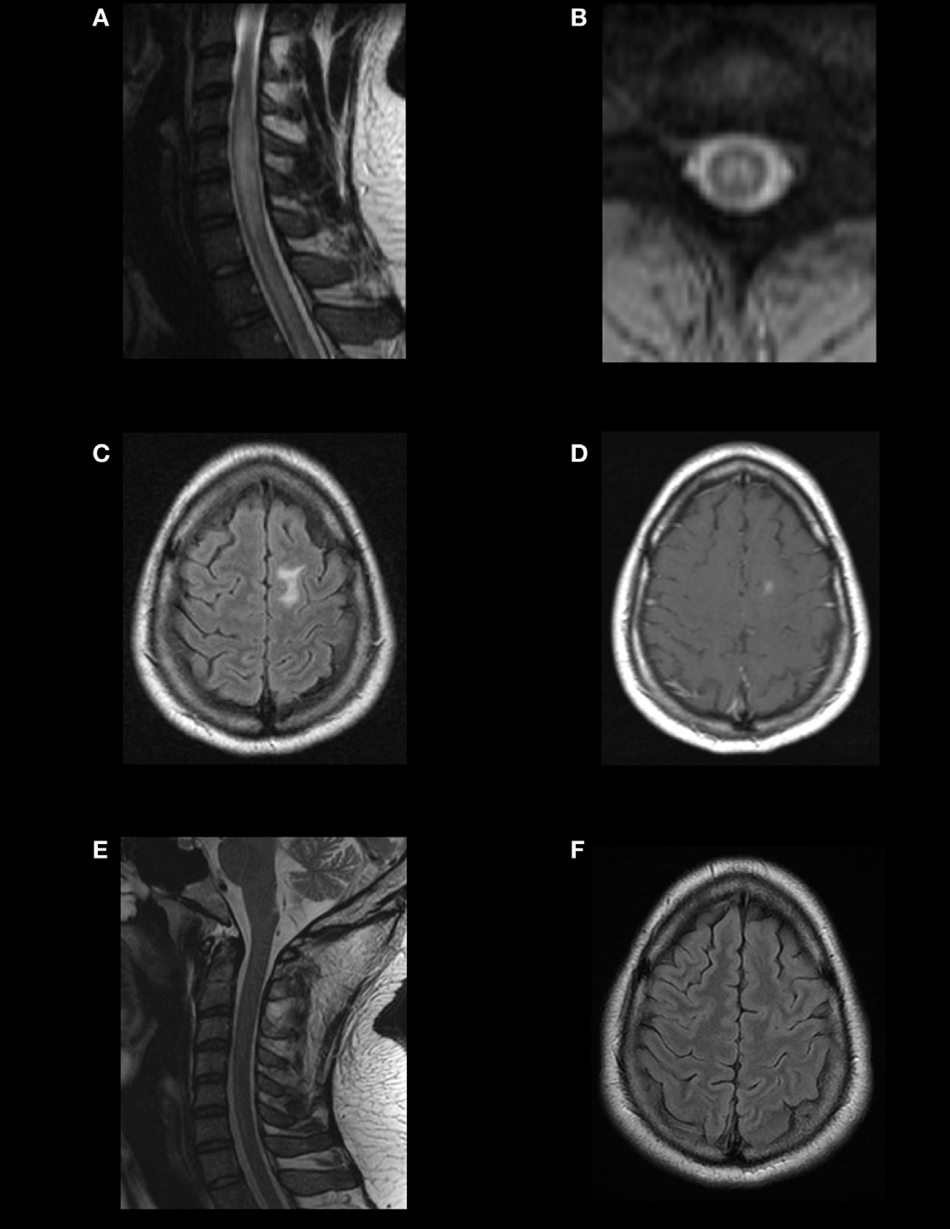
Magnetic resonance imaging (MRI) findings and evolution in a MOG-IgG-positive case fulfilling the diagnosis of neuromyelitis optica spectrum disorder (NMOSD). A young adult presented with recurrent optic neuritis followed by transverse myelitis. Spinal MRI showed a T2-hyperintense, centrally located longitudinally extensive transverse myelitis extending from C4 to C7, with mild cord swelling **(A,B)**. Brain MRI showed a T2/FLAIR-hyperintense lesion on the left superior frontal gyrus **(C)**, with gadolinium enhancement **(D)**. In cell-based assays, aquaporin-4 immunoglobulin G (AQP4-IgG) was negative, and MOG-IgG was positive. Response to immunotherapy was excellent. Follow-up MRI showed complete resolution of the brain and spinal lesions **(E,F)**. This case illustrates that the imaging patterns of MOG-IgG-associated ON, encephalitis, and myelitis (MONEM) often overlap with those of NMOSD, and some cases may even fulfill the 2015 criteria for the diagnosis of NMOSD without AQP4-IgG.

The search for novel biomarkers among AQP4-IgG-seronegative patients revealed the presence of MOG-IgG in a subset of such patients (Figure 2). The demographic, clinical, and paraclinical features of MOG-IgG in comparison to AQP4-IgG NMOSD have been described by a number of studies (48–51). Notably, similar features have been reported across independent cohorts of different ethnic backgrounds and geographical areas, thus indicating the consistency of the findings. The main features that distinguish MOG-IgG from AQP4-IgG NMOSD are summarized in Table 1.

**Table 1:** Features suggestive of MOG-IgG as opposed to aquaporin-4 immunoglobulin G in neuromyelitis optica spectrum disorder.

Male gender
Caucasian ethnicity
Single attack or only a few attacks
Bilateral or recurrent optic neuritis sparing the optic chiasm
Longitudinally extensive transverse myelitis involving the lumbar segment and *conus medullaris*
Good recovery after attacks

### Prevalence

The prevalence of MOG-IgG seropositivity among patients with NMOSD or limited forms has been reported by several studies and varies widely, depending mainly on each sample’s inclusion criteria (for example, children and/or adults; diagnosed based on 2006 criteria for NMO or 2015 criteria for NMOSD, etc.) but also on the detection technique used in each study. A summary of the reported prevalence rates is provided in Table 2.

**Table 2:** Reported prevalence rates of MOG-IgG seropositivity (based on cell-based assays) among patients with neuromyelitis optica spectrum disorder (NMOSD) and limited/related forms, according to phenotype and aquaporin-4 immunoglobulin G (AQP4-IgG) status.

Reference	Sample description	Prevalence of MOG-IgG seropositivity		
		Irrespective of AQP4-IgG status	Among AQP4-IgG seronegative	Among AQP4-IgG seropositive
Mader et al. (9)	Definite neuromyelitis optica (NMO)	3/45 (6.7%)	2/2 (100%)	1/45 (2.2%)
	High-risk NMO [longitudinally extensive transverse myelitis (LETM) or recurrent ON]	7/53 (13.2%)	7/21 (33.3%)	0/32 (0%)
Kitley et al. (8)	NMO/NMOSD	4/71 (5.6%)	4/27 (14.8%)	0/44 (0%)
Rostasy et al. (52)	Children with recurrent ON	12/15 (80%)	12/15 (80%)	0/0 (0%)
Rostásy et al. (53)	Children with definite NMO	3/8 (37.5%)	3/6 (50%)	0/2 (0%)
Höftberger et al. (50)	Definite NMO	6/48 (12.5%)	4/9 (44.4%)	2/39 (5.1%)
	LETM	5/84 (6%)	5/68 (7.4%)	0/16 (0%)
	Severe, bilateral, or recurrent ON	7/39 (17.9%)	7/33 (21,2%)	0/6 (0%)
Ramanathan et al. (54)	AQP4-IgG-seronegative NMO/NMOSD	N/A	9/23 (39.1%)	N/A
Sato et al. (48)	Definite NMO	1/101 (1%)	1/16 (6.2%)	0/85 (0%)
	LETM	5/78 (6.4%)	5/35 (14.3%)	0/43 (0%)
	Bilateral or recurrent ON	10/36 (27.8%)	10/25 (40%)	0/11 (0%)
Chalmoukou et al. (55)	AQP4-seronegative ON (uni- or bilateral, monophasic, or recurrent)	N/A	8/111 (7.2%)	N/A
Cobo-Calvo et al. (56)	AQP4-IgG-seronegative LETM	N/A	13/56 (23.2%)	N/A
Hyun et al. (57)	Isolated LETM	4/108 (3.7%)	4/53 (7.5%)	0/55 (0%)
Kim et al. (58)	Definite NMO	0/23 (0%)	0/3 (0%)	0/20 (0%)
	Recurrent or bilateral ON	10/30 (33.3%)	10/25 (40%)	0/5 (0%)
Pröbstel et al. (59)	NMO/NMOSD	4/48 (8.3%)	4/17 (23.5%)	0/31 (0%)
Siritho et al. (60)	AQP4-seronegative idiopathic inflammatory central nervous system diseases with a non-multiple sclerosis phenotype	N/A	6/29 (20.7%)	N/A
Yan et al. (61)	NMOSD	24/125 (19.2%)	14/46 (30.4%)	10/79 (12.7%)
van Pelt et al. (51)	NMOSD or limited forms	N/A	20/61 (32.8%)	N/A

*N/A, not available*.

We found that 7.4% of all NMOSD patients were seropositive for MOG-IgG, while 64.7% were seropositive for AQP4-IgG (48). Kim et al. reported that 6.3% of patients with inflammatory demyelinating diseases of the CNS had MOG-IgG, while 18.1% had AQP4-IgG (58). Initially, the prevalence of MOG-IgG might seem quite low in comparison to that of AQP4-IgG in NMOSD. However, when adjustments are made for patients with specific phenotypes and those who lack AQP4-IgG, the proportion of MOG-IgG-seropositive patients becomes quite higher. Two recent studies reported that 40% of patients with bilateral or recurrent ON and negative AQP4-IgG were positive for MOG-IgG (48, 58). In regard to AQP4-IgG-seronegative LETM, the reported prevalence of MOG-IgG has ranged between 7.4 and 23.2% (48, 50, 56, 57). Finally, among children, that prevalence seems to be even higher: 50% for patients with definite NMO (53) and 80% for patients with recurrent ON (52).

Notably, double positivity (i.e., for both AQP4-IgG and MOG-IgG) is usually not expected or found when CBA are used, which likely suggests that each antibody is present in distinct disease processes. The isolated cases reported to have double seropositivity are extremely rare and usually have significantly higher relapse rates, residual disability, and magnetic resonance imaging (MRI) lesion burden, but these characteristics are compatible with AQP4-IgG-seropositive NMOSD (43, 61, 62).

### Demographic Features

The proportion of males is generally higher (between 47 and 62%) among people with MONEM than among those with AQP4-IgG NMOSD (only 10–15%) (46, 48–51, 63). However, a recent multicenter European study reported that 74% of MOG-IgG-seropositive patients were female, resulting in a female-to-male ratio of 2.8:1 (11). Nonetheless, even this degree of female predominance is low in comparison to the ratio of up to 9:1 described in AQP4-IgG patients (64).

The proportion of Caucasian ethnicity is usually deemed as higher in MONEM (78–90 versus 60–63% in AQP4-IgG NMOSD) (49, 51), but a recent study reported no ethnic bias (12). Regarding age at onset, some studies report younger age (around the third decade) in the MOG-IgG group versus the AQP4-IgG group (the fourth decade) (49, 50), while others report no difference (48).

### Clinical Phenotypes

Two independent groups have reported similar findings regarding the clinical phenotypes associated with MONEM: ON was the leading phenotype (41–63%), followed by LETM (29–31%), NMO (6–24%), and encephalomyelitis (2–6%), while in AQP4-IgG patients, the reported phenotypes were NMO in approximately 60%, LETM in approximately 30%, and ON in approximately 10% (10, 11, 48, 50).

Some series have reported that the simultaneous occurrence of ON and TM is more common among MOG-IgG patients than among AQP4-IgG patients (49–51), but another series reported the opposite (48). Interestingly, monophasic presentation with both ON and TM occurring during the same attack, which resembles the original case of NMO described by Devic in 1894, is likely more common in MONEM than in AQP4-IgG NMOSD.

The first attack of TM or ON can be severe. In the largest series, visual acuity was reported to be <0.1 at least once in 69% of patients with ON (11). Among patients with TM, motor symptoms were frequent and included tetraparesis in 28%, paraparesis in 48%, and severe weakness (British Medical Research Council grades ≤2) in 21% (11).

Brainstem involvement is also seen in MONEM. The area postrema syndrome (persistent nausea, vomiting, or hiccups), which is usually regarded as a typical attack of NMOSD (frequency around 40%), has been reported in 6–15% of MOG-IgG-seropositive patients as well (12, 48). In another series, brainstem involvement (comprising a wide range of symptoms and signs and/or radiological findings) occurred at some point in the disease course in 30% of patients with MOG-IgG-seropositive ON and/or TM (65).

In the largest cohort of MOG-IgG-seropositive patients published so far, ADEM (or an ADEM-like episode) has been reported as the initial presentation in 18%, with most of these attacks occurring in the pediatric age range (12). More recently, cortical encephalitis has been described in MOG-IgG-positive patients, while this phenotype has not been described in AQP4-IgG NMOSD (16–20).

Finally, MOG-IgG has also been reported as positive (with proper CBA) in a subgroup of patients diagnosed with MS (66). Nonetheless, their clinical pictures were actually atypical for MS: severe attacks of myelitis, ON, and/or brainstem syndromes, with failure to several disease-modifying drugs. Therefore, it is likely that the diagnosis in this subgroup should be revised to MONEM, rather than MS, despite the fact that they reportedly fulfilled the MRI criteria for MS.

### Disease Course

A preceding infectious prodrome has been reported in 47% of the cases. For instance, Amano et al. described LETM after influenza infection (67) and Nakamura et al. reported anti-MOG-IgG ADEM presentation after infectious mononucleosis (68). Jarius et al. also reported two cases following vaccination (69).

Different series, with median follow-up times ranging from 12 to 24 months, suggest a higher proportion of single attacks in MONEM (41–70%) than in AQP4-IgG NMOSD (7–29%) (48, 50, 51). Nevertheless, relapses do occur in MOG-IgG-seropositive patients, but the number of relapses is usually lower in this group (48, 49, 51). With long-term follow-up, the proportion of patients with a single attack is likely reduced, as illustrated by one study with a longer median follow-up time (43 months), in which the proportion of patients with a single attack was only 29% (10). Moreover, in a cohort of incident cases (i.e., wherein the detection of MOG-IgG was made shortly after onset and before the second relapse), the proportion of relapsing cases after a median follow-up time of 16 months was 36%, with an annualized relapse rate (ARR) of 0.2 in those who were followed-up for ≥24 years (12).

Pröbstel et al. reported that the median time until a second attack was longer in MOG-IgG-seropositive patients (11.3 years) than in AQP4-IgG-seropositive (3.2 years) or double seronegative (3.4 years) patients (70). In the series by Jarius et al., the median time between the first and second attacks in MOG-IgG-seropositive patients was 5 months, although the interval could be longer (more than 12 months in eight patients and up to 492 months in one patient) (11).

### Radiological Features

On brain MRI, lesions involving the deep gray matter and lesions adjacent to the fourth ventricle were found to be more frequent in AQP4-IgG NMOSD than in MONEM (49). In one study, supratentorial brain lesions were seen in 35% of MOG-IgG patients at disease onset and in 47% at last follow-up; infratentorial abnormalities were present in 15% at onset and in 29% at last follow-up (11).

On orbital MRI, optic nerve head swelling, retrobulbar involvement, and contrast-enhancing lesions of the optic nerves with perineural enhancement were significantly more frequent in ON associated with MONEM than with AQP4-IgG NMOSD, whereas chiasmal involvement was more frequent in patients with AQP4-IgG (71, 72). There is some controversy regarding whether bilateral ON is more commonly associated with MOG-IgG or AQP4-IgG (71).

Regarding spinal cord MRI, while patients with AQP4-IgG usually present cervical (with or without brainstem involvement) and thoracic lesions, patients with MOG-IgG may present lesions of the lower cord, including the *conus medullaris* (48, 49). As with AQP4-IgG NMOSD, not all cases of MOG-IgG-associated TM are longitudinally extensive. A small proportion (7%) of MOG-IgG-seropositive patients were reported to present with short myelitis occurring after an initial episode of LETM, isolated at disease onset, or following previous episodes of ON (which could initially suggest MS) (10).

### CSF Features

CSF white cell count is usually elevated, ranging between 3 and 306 in two series, with lymphocytic predominance (11, 49). CSF pleocytosis was more frequent (92 versus 45%) in MOG-IgG-seropositive patients with a first episode of LETM than in double seronegative patients (56).

Evidence of intrathecal synthesis, assessed by the IgG index, was generally absent, suggesting that MOG-IgG is likely produced in the periphery (69). Positivity for MOG-IgG in the CSF was found in 71% of patients who were MOG-IgG-seropositive, with a median CSF MOG-IgG titer of 1:4, lower than the serum titer (69).

### Coexisting Autoimmunity

Some studies have suggested other autoimmune abnormalities to be less common among those with MOG-IgG. Specifically, antinuclear antibodies were found in only 7% of MOG-IgG patients (versus 43% of AQP4-IgG patients) (48), and coexisting autoimmune conditions were reported in only 11% of MOG-IgG individuals (versus 45% of AQP4-IgG subjects) in another series (49). On the other hand, by using a wider panel of autoantibodies, Jarius et al. reported coexisting autoantibodies in 42% of MOG-IgG-seropositive patients, while concomitant autoimmune disorders were present in only 8% of them (11).

### Prognosis

Recovery from attacks is usually reported as better in MONEM than in AQP4-IgG-seropositive NMOSD. In our experience, the degree of improvement after an attack, measured by the Expanded Disability Status Scale (EDSS) score and visual acuity, was better for MOG-IgG-seropositive patients (48) than for others. In the series by Kitley et al., the median decrease in EDSS scores between episode onset and recovery was greater in MOG-IgG-seropositive patients than in AQP4-IgG-seropositive patients (6 points and 2 points, respectively), despite similar EDSS scores during the onset episode; moreover, the risks for residual visual and motor disability were lower in patients with MOG-IgG (49).

Overall, MONEM patients with ON seem to present a much lower risk of severe and sustained visual impairment than AQP4-IgG-seropositive patients (71). Some studies have used optic coherence tomography to compare these two groups in terms of measurements of the ganglion cell-inner plexiform layer and the retinal nerve fiber layer thickness. They suggested that a single episode of ON may be associated with milder retinal neuronal loss in MONEM than in AQP4-IgG-seropositive NMOSD, despite more severe optic nerve swelling on presentation in the former (72–74). On the other hand, one of these studies also reported a higher frequency of ON relapses in MONEM, in such a way that an increased number of episodes ended up leading to a degree of retinal layers thinning similar to that seen in AQP4-IgG-seropositive NMOSD (73).

In patients with LETM who were seronegative for AQP4-IgG, those who had MOG-IgG presented a higher degree of recovery after attacks but had a higher predisposition to subsequent ON than those who were MOG-IgG seronegative (56).

In comparison to both AQP4-IgG-seropositive patients and those who are double seronegative, patients with MOG-IgG usually reported to have a better overall outcome (48, 50, 51, 75). However, as mentioned previously, severe disability after ON or LETM does occur in MOG-IgG-seropositive patients, meaning that not all individuals will have a full recovery (48). In a large cohort of MOG-IgG-seropositive cases, followed-up for a median of 28 months, 28% were left with permanent bladder dysfunction; 21% (among males) with erectile dysfunction; 20% with bowel dysfunction; 16% with visual acuity <6/36 in at least one eye; and 5% with EDSS score ≥6 (12).

## MOG-IgG in Pediatric Patients

Several clinical syndromes compatible with MONEM have been described in pediatric patients with MOG-IgG seropositivity, mainly multiphasic ADEM, ADEM followed by ON, recurrent ON, TM, and AQP4-IgG-seronegative NMOSD (76). Previous studies associated the presence of MOG-IgG with MS in children younger than 10 years, but this association was not consistent in adult patients. However, recent findings suggest that the presence of MOG-IgG could also predict a non-MS disease course in this age group (77, 78). As the MOG-IgG titers found in MS are usually lower and different MS diagnostic criteria were used in various studies (78), there is no clear association between MOG-IgG and MS, even in pediatric patients. Similarly, some MOG-IgG-seropositive/AQP4-IgG-seronegative patients diagnosed with NMO do not fulfill the newly revised diagnostic criteria for NMOSD (1).

Although MOG-IgG seropositivity apparently does not predict the initial clinical presentation or the disease course (monophasic or relapsing), some studies have demonstrated that MOG-IgG-seropositive patients present different clinical manifestations according to age (77, 79). Fenandez-Carbonell et al. found a bimodal distribution in 13 pediatric MOG-IgG-seropositive patients, with encephalopathy being more common in younger patients (4–8 years) and ON in older patients (13–18 years) (79).

Concerning the evolution of MOG-IgG seropositivity, some patients, mainly those presenting with ADEM with full recovery, lost their positivity after the acute demyelinating episode (80), whereas others persisted with detectable titers suggesting chronic inflammation (70). Mayer et al. evaluated the ability to maintain IgG seropositivity to vaccines in patients who have lost their MOG-IgG-seropositivity and found that MOG-IgG-secreting cells are less competent to seed the survival niches than those related to immunity to measles and rubella (81). The immunological mechanisms of this phenomenon remain to be elucidated. The presence of MOG-IgG in ADEM could be due to the recognition of viral or bacterial antigens similar to MOG (cross-reacting immune response) (70).

These findings indicate that MONEM is a continuum of CNS inflammatory demyelinating diseases also found in pediatric patients and that the maintenance of seropositivity could predict its clinical evolution. Long-term studies are needed to assess the value of MOG-IgG as a biomarker, understand the susceptibility of this age group and observe the outcomes of the demyelinating episodes in the developing CNS.

## Treatment of MONEM

The management of acute attacks usually includes the same strategies used for other CNS immune-mediated conditions, such as oral or intravenous methylprednisolone (IVMP), plasma exchange (82), intravenous immunoglobulin (IVIg), and cyclophosphamide (83). In a study by our group, 87% of these patients achieved good recovery with IVMP (48). In a series of eight patients with idiopathic ON (i.e., ON patients who did not meet the criteria for NMOSD, MS, or other diseases) who were found to be seropositive for MOG-IgG, seven had a good or complete response to IVMP in the acute phase, and only two presented relapses (84). There are also a few reports of cases successfully treated by lymphocytapheresis after failure of initial therapies (85, 86).

The greatest challenge is long-term management, since the available pharmacological options for NMOSD (corticosteroids, immunosuppressive agents, rituximab, and plasma exchange) have not been specifically assessed in the subset of patients with MONEM (83).

Actually, it is still uncertain whether all MONEM patients need long-term treatment, given the possibility of a monophasic course, the usually lower relapse rate and the generally good recovery after attacks in response to acute treatment. In the series by Höftberger et al., chronic therapy was used in only one-third of the MOG-IgG-seropositive patients versus nearly all of the AQP4-IgG-seropositive NMOSD patients (50). On the other hand, treatment with immunosuppressive drugs for at least 3 months following the onset attack has been associated with reduced risk of a second relapse, in a large series (12).

When chronic treatment is indicated, it seems that first-line immunosuppressive drugs may reduce the number of relapses in a considerable proportion of patients. MOG-IgG has been found in a subset of patients with the chronic relapsing inflammatory ON phenotype, which is responsive to corticosteroids; these patients can usually be managed with a low-dose oral regimen (55). Some authors propose that AQP4-IgG-seronegative NMOSD be treated with azathioprine as initial therapy, with escalation to mycophenolate mofetil or rituximab as required (7); whether the same approach holds valid for MONEM is yet to be determined.

Jarius et al. reported some experience with long-term immunosuppressant agents in MOG-IgG-seropositive patients (11). The mean ARR for azathioprine was 0.99, with 41% of the attacks occurring during the first 6 months, and most of these early attacks occurred in individuals who were not under co-treatment with corticosteroids (11). For methotrexate, the mean ARR was 0.22, which is lower than the mean ARR of 0.95 found among all patients in this series (11). With rituximab, three out of nine patients experienced a decline in the ARR; in the remaining patients, most relapses were observed either shortly after rituximab infusion or at the end-of-dose period (11).

Another observational study demonstrated a beneficial effect of chronic immunotherapy in reducing the ARR in MONEM. The lowest treatment failure rates were seen with long-term oral prednisolone (5%) and rituximab (17%) (13). Maintenance IVIg and mycophenolate mofetil were associated with failure rates of 43 and 44%, respectively; yet, they too were partially effective in reducing the pretreatment ARR (13).

While MONEM belongs to the group of demyelinating disorders, that includes MS, it has some characteristics of autoimmunity, with autoantibody production as seen in AQP4-IgG-seropositive NMOSD; therefore, its response to MS therapies is unpredictable. Many MS therapies may actually exacerbate AQP4-IgG-seropositive NMOSD, as has been reported for beta-interferons (87, 88), natalizumab (89, 90), and fingolimod (91, 92). Mitoxantrone and natalizumab have failed to reduce relapses in patients with MOG-IgG (11).

Currently, it remains to be determined whether the course of the disease (i.e., single attack or further relapses) or higher titers or persistence of MOG-IgG can predict the need for long-term therapy. Upcoming therapeutic trials of NMOSD should enroll or stratify patients according to the presence or absence of both AQP4-IgG and MOG-IgG.

## Conclusion

Despite some overlap, MONEM exhibits different pathophysiological and phenotypic features than both AQP4-IgG-associated NMOSD and typical MS. In addition, the clinical spectrum of MONEM expands beyond NMOSD, likely including ADEM and other demyelinating syndromes. We believe that MOG-IgG and AQP4-IgG should be considered biomarkers of different disease processes instead of different biomarkers for the same condition. It is possible that MOG-IgG-associated “NMOSD” is not truly a part of the NMO spectrum; instead, MONEM could represent another disorder that may overlap in certain clinical phenotypes. Further research on the pathophysiology of MONEM and the development of clinical trials that treat MONEM patients as a specific study population are warranted to enable the development of evidence-based management strategies for these patients.

## Author Contributions

GP and LO were responsible for acquisition, analysis, and interpretation of data, and drafted the manuscript content. BC drafted the manuscript content. SA-P drafted the manuscript content. DC drafted the manuscript content, helped in administrative, technical, and material support. KF had full access to all of the data in the study and takes responsibility for the integrity of the data and the accuracy of the data analysis, critical revision of the manuscript and did study supervision. DS had full access to all of the data in the study and takes responsibility for the integrity of the data and the accuracy of the data analysis, critical revision of the manuscript and did study supervision. GP and LO contributed equally.

## Conflict of Interest Statement

GP: scholarship from World Federation of Neurology, ECTRIMS, Novartis. LO: scholarship from FAPESP/Brasil. BC: scholarship from CNPq/Brasil. SA-P: nothing to disclose. DC: Research support from CAPES/Brasil (CSF-PAJT—88887.091277/2014-00). KF: serves on scientific advisory boards for Bayer Schering Pharma, Biogen Idec, Mitsubishi Tanabe Pharma Corporation, Novartis Pharma, Chugai Pharmaceutical, Ono Pharmaceutical, Nihon Pharmaceutical, Merck Serono, Alexion Pharmaceuticals, Medimmune, and Medical Review; has received travel funding and speaker honoraria from Bayer Schering Pharma, Biogen Idec, Eisai Inc., Mitsubishi Tanabe Pharma Corporation, Novartis Pharma, Astellas Pharma Inc., Takeda Pharmaceutical Company Limited, Asahi Kasei Medical Co., Daiichi Sankyo, and Nihon Pharmaceutical; serves as an editorial board member of Clinical and Experimental Neuroimmunology (2009–present) and an advisory board member of *Sri Lanka Journal of Neurology*; has received research support from Bayer Schering Pharma, Biogen Idec Japan, Asahi Kasei Medical, The Chemo-Sero-Therapeutic Research Institute, Teva Pharmaceutical, Mitsubishi Tanabe Pharma, Teijin Pharma, Chugai Pharmaceutical, Ono Pharmaceutical, Nihon Pharmaceutical, and Genzyme Japan; and is funded by Grants-in-Aid for Scientific Research from the Ministry of Education, Culture, Sports, Science and Technology of Japan (#22229008, 2010-2015;#26293205, 2014-2016) and by Grants-in-Aid for Scientific Research from the Ministry of Health, Welfare and Labor of Japan (2010–present). DS has received a scholarship from the Ministry of Education, Culture, Sports, Science and Technology (MEXT) of Japan; a Grants-in-Aid for Scientific Research from the Japan Society for the Promotion of Science (KAKENHI 15K19472); research support from CNPq/Brasil (425331/2016-4), TEVA (research grant for EMOCEMP Investigator Initiated Study – NCT no. 61080516.4.1001.5336), and Euroimmun AG (Neuroimmunological Complications associated with Arboviruses); and speaker honoraria from Biogen, Novartis, Genzyme, TEVA, Merck-Serono, Roche, Bayer and is an advisory board member of Shire, Roche, TEVA, Merck-Serono and Quest/Athena Diagnostics.
